# A Comprehensive Diverse ‘-omics’ Approach to Better Understanding the Molecular Pathomechanisms of Down Syndrome

**DOI:** 10.3390/brainsci7040044

**Published:** 2017-04-21

**Authors:** Keiichi Ishihara, Satoshi Akiba

**Affiliations:** Department of Pathological Biochemistry, Division of Pathological Science, Kyoto Pharmaceutical University, 5 Misasagi Nakauchi-cho, Ymashina-ku, Kyoto 607-8414, Japan; akiba@mb.kyoto-phu.ac.jp

**Keywords:** proteomics, transcriptomics, Down syndrome, mouse models

## Abstract

Diverse ‘-omics’ technologies permit the comprehensive quantitative profiling of a variety of biological molecules. Comparative ‘-omics’ analyses, such as transcriptomics and proteomics, are powerful and useful tools for unraveling the molecular pathomechanisms of various diseases. As enhanced oxidative stress has been demonstrated in humans and mice with Down syndrome (DS), a redox proteomic analysis is useful for understanding how enhanced oxidative stress aggravates the state of individuals with oxidative stress-related disorders. In this review, ‘-omics’ analyses in humans with DS and mouse models of DS are summarized, and the molecular dissection of this syndrome is discussed.

## 1. Introduction

**Figure 1:**
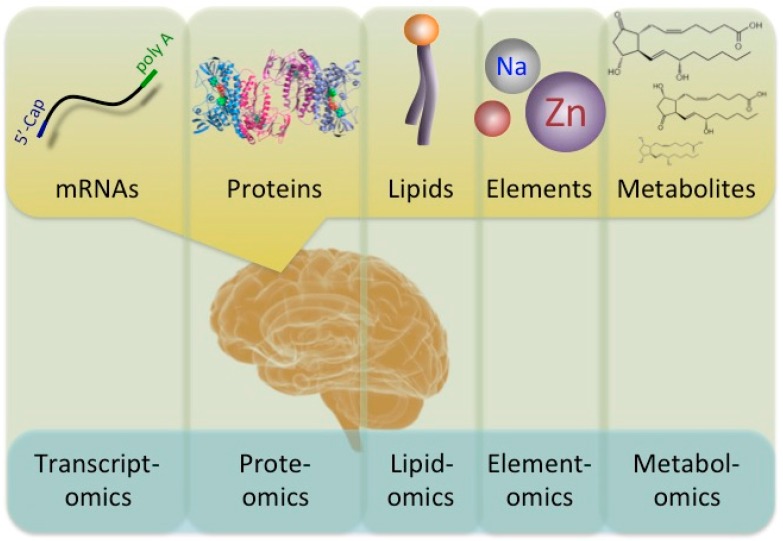
A diagram of the interaction between ‘-omics’ and the flow of biological molecules. A number of ‘-omics’ analyses have been developed. Transcriptomics, proteomics, lipidomics, elementomics, and metabolomics analyses describe the levels of mRNA, proteins, lipids, such as phospholipids, elements, such as trace biometals, and various metabolites, such as prostaglandins, respectively.

Understanding the changes in molecules provides important and valuable information for evaluating the molecular pathomechanisms of diseases. Diverse ‘-omics’ technologies permit the quantitative monitoring of various biological molecules in a comprehensive manner. Comprehensive expression profiles are designated by adding the suffix ‘-omics’ onto previously used terms, such as prote-omics (protein), transcript-omics (transcripts), lipid-omics (lipid), metabol-omics (metabolites), and so on (Figure 1). Comparative ‘-omics’ analyses comparing the profiles of healthy subjects and populations with some illness are a powerful and useful approach for better understanding molecular pathomechanisms. Indeed, a variety of disorders, including neurological disorders, have been subjected to ‘-omics’ analyses.

Although most ‘-omics’ analyses assess the levels of target molecules, protein, transcripts, fatty acids, and various metabolites in organs, redox proteomics and phospho-proteomics indicate the degree of oxidative stress and activation of signal transduction or transcriptional factors, respectively. Thus, proteomics can be used to determine not only the protein expression, but also the degree of post-translational modifications, such as phosphorylation and oxidation. However, no single ‘-omics’ analysis can fully unravel the complexities of some disorders. Therefore, the integration of multiple layers of information—the multi-’-omics’ approach—is required to acquire a precise picture of the pathophysiology of a given disease.

**Figure 2:**
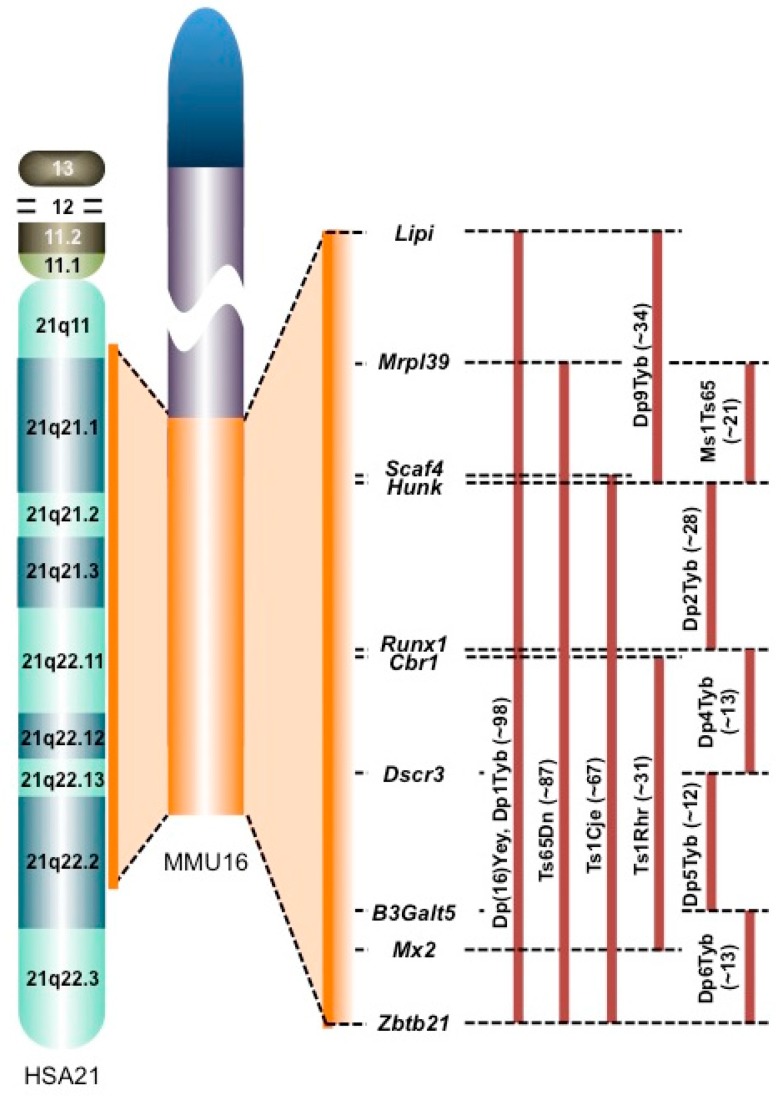
Segmentally trisomic regions of DS mouse models. Most of HSA21 is syntenic to the distal end of MMU16. The trisomic regions in several mouse models of DS are compared on the right of the MMU16 (orange bars). The numbers in brackets represent the number of protein-coding genes within each MMU16 region according to Ensembl release 87. *Lipi*: lipase, member I, *Mrpl39*: mitochondrial ribosomal protein L39, *Scaf4*: SR-related CTD-associated factor 4, *Hunk*: hormonally upregulated Neu-associated kinase, *Runx1*: runt-related transcription factor 1, *Cbr1*: carbonyl reductase 1, *Dscr3*: Down syndrome critical region gene 3, *B3Galt5*: UDP-Gal:betaGlcNAc beta 1,3-galactosyltransferase, polypeptide 5, *Mx2*: MX dynamin-like GTPase 2, and *Zbtb21*: zinc finger and BTB domain containing 21.

In this review, we discuss the changes in the molecules in brains from individuals with Down syndrome (DS) and DS mouse models according to ‘-omics’ analyses. Although DS is caused by trisomy of human chromosome 21 (HSA21), the distal end of mouse chromosome 16 (MMU16) and part of MMU10 and MMU17 share synteny with human chromosome 21 (HSA21) [1] (Figure 2). Therefore, several mouse models carrying an extra partial MMU16, MMU10, or MMU17 have been established as mouse models for DS (Figure 2). In particular, MMU16 has synteny with a large portion of HSA21, which has led to the establishment of several mouse models of DS: Dp(16)Yey [2], Ts65Dn [3], Ts1Cje [4], Ms1Ts65 [5], and Ts1Rhr mice [6] (Figure 2). Furthermore, seven mouse models of DS carrying three copies of various segments of MMU16 syntenic to HSA21 have been recently established: Dp1Tyb, Dp2Tyb, Dp3Tyb, Dp4Tyb, Dp5Tyb, Dp6Tyb, and Dp9Tyb [7] (Figure 2). The Ts65Dn model is the most intensively studied model of DS to date and carries a 13.2 Mb segmental trisomy of MMU16, and the Ts1Cje model carrying a shorter segmental trisomy (7.6 Mb) is also widely used. Both models display DS-like phenotypes, including learning and memory deficits and/or an abnormal neuron morphology and function [3,4,8,9,10].

## 2. Comparative Transcriptomics in the DS Brain

The analysis of the expression of transcripts in humans with DS and mouse models of DS provides valuable information for understanding the pathophysiological mechanisms underlying this disease. However, whether or not the expression of trisomic genes is upregulated in a gene-dose-dependent manner and the effect of trisomic gene expression on the expression of transcripts from euploid genes remain unclear.

A number of comprehensive gene expression analyses using tissue of humans with DS and mouse models of DS have been performed. The findings from studies using brain tissue are summarized in Table 1. Although relatively few studies have been performed with human DS samples, two included in Table 1 indicate that a gene-dose-dependent increase in transcription was detected in the brain both before and after birth [11,12].

A number of comparative transcriptomic analyses in brains of mouse models of DS have been performed (Table 1). The precise brain region and the age of the samples analyzed must be considered for an accurate interpretation of these comparative transcriptomics data. However, a variety of samples have been used, and most (but not all) transcriptomic analyses comparing trisomic and euploid tissues support the hypothesis of increased mRNA levels in a gene-dose-dependent manner. In addition, a number of reports have shown that the mRNA expression of some euploid genes is disturbed in the brain of DS model mice.

Although transcriptomic analyses using DS brains in humans and mice provide important information, as described above, the interpretation of the data is very complex. RNA sequencing is a powerful analytical tool that is expected to replace microarrays for transcriptomics profiling [25]. RNA sequencing has several advantages over microarrays, such as the avoidance cross-hybridization, the limited detection range of individual probes, and non-specific hybridization. RNA sequencing could, therefore, provide new information on the gene expression in brains with DS.

## 3. Comparative Proteomics in the DS Brain

The protein expression does not always correlate with the expression of mRNA, so a comprehensive analysis of the protein expression in brains with DS is very important for understanding the pathomechanisms of the disease. A number of comparative proteomics analyses using the brains from patients and mouse models have been performed. The reports including such analyses for understanding changes in the protein expression in the brains with DS and neuronal cells derived from embryonic stem (ES) cells with an extra HSA21 are listed in Table 2. In contrast to the findings on gene expression analyses, a number of proteomics analyses failed to detect any increased expression of trisomic genes at the protein level (Table 2). This might be due to the sensitivity of the proteomic analysis, especially two-dimensional (2D)-electrophoresis, since antibody arrays successfully detected an increase in the protein expression of trisomic genes [26]. Recently-developed chemical labeling techniques using isobaric tags (i.e., tags with the same molecular weight) for the relative and absolute quantification (iTRAQ) [27] and tandem mass tags (TMT) [28] are expected to resolve the issue of the low sensitivity of separation on 2D-electrophoresis.

A number of proteins associated with proteolysis, energy metabolism, the cytoskeleton, and cell proliferation that are coded in disomic genes were found to be dysregulated in humans and mice with DS (Table 2) [29,30,31], although proteins, which were differentially expressed in both human and mouse brain with DS, have not yet been identified in these proteomic analysis. For instance, ubiquitin carboxyl-terminal hydrolase isozyme L1 (Uchl1) coded on an euploid gene showed a decreased expression in the brain of a 141G6 mouse carrying a yeast artificial chromosome (YAC), which contains HSA21 genes, Down syndrome critical region gene 3 (*Dscr3*), phosphatidylinositol glycan anchor biosynthesis, class P (*Pigp*), ripply transcriptional repressor 3 (*Ripply3*), and tetratricopeptide repeat domain 3 (*Ttc3*), as well as neurons differentiated from ES cells carrying an extra HSA21 [32,31]. In addition, proteolysis and energy metabolism-related proteins, such as Uchl1 and α-enolase, show not only differential expression, but also oxidative modification under DS conditions, as will be explain in the next section.

## 4. Redox Proteomics in the DS Brain

Since proteins are major targets of reactive oxygen, nitrogen species and aldehyde products of lipid peroxidation (LPO), comparative analysis of these protein modifications reveals the degree of oxidative stress, and the certain target molecules of enhanced oxidative stress. Therefore, redox proteomics is a useful tool to decipher the effect of oxidative stress in the brain with DS. As shown in Table 3, redox proteomics detecting carbomylated- or 4-hydroxynonenal (4-HNE) adducted-proteins revealed that the carbonylated UchL1 level was increased in the human DS brain [33]. Furthermore, although the levels of LPO-related modified proteins, such as 13(S)-hydroperoxyoctadeca-9Z,11E-dienoic acid (13-HPODE)-adducted α-enolase, glycolytic enzyme, and 4-HNE-adducted neuron specific enolase, are increased in Ts1Cje mice [34], the expression of α-enolase and neuron-specific enolase decreased in the human DS cortex and the hippocampus of 141G6 mice [29,32]. The impairment of the energy metabolism is suggested to be closely related to several age-related neurodegenerative disorders, such as Alzheimer’s disease and mild cognitive impairment (MCI) [35]. Thus, the oxidative stress-mediated decline in certain protein functions may be associated with abnormalities of the DS brain.

## 5. Other ‘-omics’ Analyses

Other ‘-omics’ analyses, such as metabolomics including lipidomics and elementomics, including metallomics, are expected to prove useful in understanding the pathophysiological state of DS. As no proteins were found to be differentially expressed in the Ts1Cje mouse brain during neonatal and postnatal life by 2D-electrophoresis-based proteomics [30], these other ‘-omics’ approaches are thought to be particularly useful.

A metabolomics approach can digest a very large volume of information regarding the levels of the complete set of low-molecular-weight molecules or metabolites synthesized by a cell. The alteration of biogenic metabolites, such as neurotransmitters and prostaglandins, in abundance provides a unique chemical fingerprint that specific cellular processes leave behind. Although there have been a number of metabolomics studies on the accumulation of such elements in the brains of Alzheimer’s disease patients (see [44]), no metabolomics studies with DS subjects have yet been reported.

Elementomics, including metallomics, is another new ‘-omics’ approach using inductively-coupled plasma-mass spectrometry (ICP-MS) [45]. Elementomics comprehensively elucidate the levels of the most common elements, including biogenic trace metals, in the tissues and cells. This approach has only been applied to study Alzheimer’s disease, with no reports in DS subjects available at present [46,47]. The dyshomeostasis of intrinsic metals, such as zinc, copper, and iron, may play a role in cognitive impairment in individuals with DS, as intersectin 1 coded in the trisomic region in DS is suggested to be involved in iron internalization. Indeed, increases in the levels of non-protein-bound iron in serum and iron-binding protein lactotransferrin in the brain have been demonstrated in individuals with DS [48,49]. Other ‘-omics’ analyses, such as metabolomic and elementomics analyses, for DS samples are expected to help unravel the pathophysiological mechanisms underlying abnormalities in the DS brain.

## Figures and Tables

**Table 1:** A comparative transcriptomic analysis of the brain with DS.

Sample (Genetic Background)	Tissue	Number of Samples	Stage	Method	Transcription of Trisomic Genes	Remarks	Reference
Human	Cerebellum	Control: *n* = 3; DS: *n* = 3	18–20 weeks of gestation	Affymetrix U133A GeneChip	A gene dosage-dependent increase in transcription was detected in the cerebellum of individuals with DS.		[11]
Human	Dorsolateral prefrontal cortex	Control: *n* = 8; DS: *n* = 7	Adult	Affymetrix human genome HG-U133A GeneChip	More than 25% of genes on HSA21 were differentially expressed, versus a median of 4.4% for all chromosomes.	Dysregulated genes are classified into development (notably Notch and Dlx family genes), lipid transport, and cellular proliferation.	[12]
Ts65Dn mice (B6Ei/C3)	Whole brain	Control: *n* = 7, 4 males, 3 males Ts65Dn: *n* = 3, 3 males	Postnatal day 30	SAGE analysis with library (28,531 tags)	The expression of most known genes from the trisomic region of mouse MMU16 in Ts65Dn is too low. Only three genes (Ifnar2, Ufngr2, and Cbr) are overexpressed in Ts65Dn males compared to control males.	It has been suggested that abnormal ribosomal biogenesis may be involved in the development and maintenance of DS phenotypes.	[13]
Ts65Dn mice (B6Ei/C3)	Cerebellum, cortex, midbrain	Control: *n* = 8 Ts65Dn: *n* = 8	Males at 1–4 months old	Quantitative real-time RT-PCR	A trend toward 1.5-fold over-expression for the trisomic genes was detected. The global over-expression level of trisomic genes in Ts65Dn was 1.44-fold in the cerebellum, 1.37-fold in the cortex, and 1.39-fold in the midbrain.		[14]
Ts65Dn mice (B6Ei/C3)	CA1 pyramidal cells	Control: *n* = 7; Ts65Dn: *n* = 9	4–9 months old	Custom-designed array (576 cDNA/ESRs)	N/A	Downregulation of neutrophins and their cognate neutrophin receptors.	[15]
Ts65Dn mice (B6Ei/C3)	Cerebellum	Control (sedentary): *n* = 4 Control (running): *n* = 4 Ts65Dn (sedentary) *n* = 4 Ts65Dn (running): *n* = 4	Females at 9–13 months old	Agilent oligonucleotide microarray (SurePrint G3 Mouse Gene Expression 8 × 60 K Microarray)	Forty tested trisomic genes showed higher expression in Ts65Dn mice than in euploid mice, with an average ratio of Ts65Dn/WT 1.47.		[16]
Ts65Dn mice (B6Ei/C3)	CA1 pyramidal cells	Control: *n* = 12 Ts65Dn: *n* = 13	10–24 months old	Custom-designed array (576 cDNA/ESRs)	N/A	Dysregulation of excitatory and inhibitory neurotransmission receptor families and neurotrophins, including brain-derived neurotrophic factor, as well as several cognate neurotrophin receptors.	[17]
Ts1Cje mice (C57BL/6J)	Fetal brain	Control: *n* = 5 Ts1Cje: *n* = 5	Embryonic day 15.5	Affymetrix mouse gene 1.0 ST arrays	About half of the trisomic genes were significantly upregulated in the embryonic brain of Ts1Cje mice.		[18]
Ts1Cje mice (C57BL/6J)	Whole brain	Control: *n* = 6 Ts1Cje: *n* = 6	Males at postnatal day 0	Affymetrix murine genome U74A and U74B microarrays	The expression of most genes in the trisomic region was increased approximately 1.5-fold, and the top 24 most consistently over-expressed genes in Ts1Cje mice were all located in the trisomic region.	The transcripts of trisomic genes were mainly overexpressed in a gene-dose-dependent manner.	[19]
Ts1Cje mice (C57BL/6)	Cerebellum	Control: *n* = 2 for each stage Ts1Cje: *n* = 2 for each stage	Postnatal day 0, 15, and 30	Affymetrix murine genome U74A version	The mean expression ratios of trisomic genes between Ts1Cje and controls were 1.66, 1.32, and 1.32 at P0, P15, and P30, respectively, whereas with euploid genes, the ratios were 1.08, 1.12, and 1.02 at P0, P15, and P30, respectively.	In the cerebellum of Ts1Cje mice, six homeobox genes and two genes belonging to the Notch pathway showed severely decreased expression	[20]
Ts1Cje mice (B6C3SnF1/ Orl)	Cerebellum	Control: *n* = 2 for each stage Ts1Cje: *n* = 2 for each stage	Postnatal day 0, 3, 7, and 10	Agilent RNA 6000	A prevailing gene dosage effect of trisomy and a limited secondary effect on postnatal development were noted. Approximately 80% of gene expression differences were attributed to dosage imbalance, suggesting that the trisomic genes are likely to be directly responsible for the phenotype present in cerebellum of Ts1Cje mice.		[21]
Ts1Cje mice (C57BL/6)	Cerebral cortexcerebellum hippocampus	Control: *n* = 3 for each stage Ts1Cje: *n* = 3 for each stage	Postnatal day 1, 15, 30, and 84	Affymetrix murine genome U74A version 3 microarray	A gene dosage-dependent increase in transcription was detected in the cerebellum of individuals with DS.	The Jak-Stat pathway may be overstimulated in the brain of Ts1Cje mice.	[22]
Ts1Cje mice (C57BL/6J)	Cerebral cortex hippocampus	Control: *n* = 5 Ts1Cje: *n* = 6	Females at 2–2.5 months old	Affymetrix mouse gene 1.0 ST arrays	Of the 77 genes present in the trisomic region of Ts1Cje mice, 22 (28.6%) were differentially regulated in either the cortex or hippocampus, while the expression of the remaining 46 (71.4%) was not affected.	Dysregulation of NFAT signaling, and G-protein signaling (e.g., olfactory perception)	[23]
Ts1Cje mice (B6C3SnF1/Orl)	Neural progenitor cells	Control: *n* = 3 Ts1Cje: *n* = 3	Neurospheres were derived from E14.5 cortex	DNA microarrays (RNG-MRC_MM25k_EVRY)	The expression ratios of 54% of trisomic genes (Ts1Cje/WT) were significantly higher than the expected diploid gene ratio of 1.0.	Ts1Cje neural progenitors proliferated at a slower rate. Some euploid genes involved in proliferation, differentiation, and the glial function were dysregulated.	[24]

**Table 2:** A comparative proteomic analysis of the brain with DS.

Sample	Tissue	Sample Information		Method	Differentially Expressed Proteins	Remarks	Reference
Human	fetal cortex	controls (18.8 ± 2.2 weeks of gestation) *n* = 7 (one female, six males) DS (19.6 ± 2.0 weeks of gestation) *n* = 9 (two females, seven males)		2D-PAGE/MALDI- TOF-MS	14-3-3γ (↓), Receptor of activated protein C kinase 1 (RACK1) (↓)		[36]
Human	fetal cortex	controls (18.8 ± 2.2 weeks of gestation) *n* = 7 (one female, six males) DS (19.6 ± 2.0 weeks of gestation) *n* = 9 (two females, seven males)		2D-PAGE/MALDI- TOF-MS	Double-strand-break repair protein rad 21 (Rad21) (↑), Eukaryotic initiation factor 3 (eIF3) p47 subunit 5 (↑), heat shock protein (Hsp) 75 (↑), septin 7 (↓), β- amyloid precursor-like protein 1 (↓), β-tubulin (↓)		[37]
Human	frontal cortex	Young controls (13.1 ± 15.3 years old) *n* = 6 Old controls (53.0 ± 8.5 years old ) *n* = 6 DS (11.01 ± 10.9 years old) *n* = 6		2D-PAGE/nano-LC-MS	DS vs. young controls Ras-related protein Rab3a (↓), Guanine nucleotide-binding protein 1 (Bnb1) (↓), Apolipoprotein E (↓), Transitional endoplasmic reticulum ATPase (Vcp) (↓), pyridoxal phosphate phosphatase (↓), Malate Dehydrogenase 2 (Mdh2) (↑), α-enolase (↓)	Overlapping and independent molecular pathways, such as energy metabolism, oxidative damage, protein synthesis, and autophagy, are suggested to be involved in DS, aging, and DA/AD.	[29]
Human	frontal cortex, cerebellum	frontal cortex controls (62.80 ± 8.61 years old) *n* = 5; AD (60.80 ±103.62 years old) *n* = 5 DS (56.00 ± 10.49 years old) *n* = 5	cerebellum controls (68.50 ± 6.25 years old) *n* = 4 AD (59.86 ± 6.47 years old) *n* = 7 DS (55.43 ±8.62 years old) *n* = 7	2D-PAGE/ MALDI-TOF-MS	frontal cortex Histamine-N-methyltransferase (↓)		[38]
Ts65Dn mice	cerebellum, cerebral cortex	Control males (4.4–7.8 months old) *n* = 6; Ts65Dn males (4.4–7.8 months old) *n* = 5		Protein arrays (64 proteins/protein modifications)	Only a small number of trisomic proteins were increased in a gene-dose-dependent manner.	Ts65Dn mice have lost the correlations seen in control mice among levels of functionally related proteins, including the components of the MAP kinase pathway and subunits of the NMDA receptor.	[39]
Ts65Dn mice	cerebellum	Control and Ts65Dn (postnatal day 0, 16, and 21 and 3, 4–6, 8, 12, and 14–21 months old) *n* = 52 (total)		2D-PAGE/MS	Carbonic anhydrase II (↑)	Increased levels of carbonic anhydrase II in the developing brain with DS	[40]
Ts65Dn mice	hippocampus	Wild-type/context-shock/saline (males at 3–4 months old) *n* = 10; wild-type/shock-context/saline (males at 3–4 months old) *n* = 10; wild-type/context-shock/memantine (males at 3–4 months old) *n* = 10; wild-type/shock-context/memantine (males at 3–4 months old) *n* = 10; Ts65Dn/context-shock/saline (males at 3–4 months old) *n* = 9; Ts65Dn/shock-context/saline (males at 3-4 m-old) *n* = 10; Ts65Dn/context-shock/memantine (males at 3–4 months old) *n* = 10; Ts65Dn/shock-context/memantine (males at 3–4 months old) *n* = 10		Reverse phase protein arrays (85 proteins/protein modifications)		(i) the dynamic responses seen in control mice in normal learning, >40% also occur in Ts65Dn in failed learning or are compensated by baseline abnormalities, and thus are considered necessary but not sufficient for successful learning, and (ii) treatment with memantine does not in general normalize the initial protein levels but instead induces direct and indirect responses in approximately half the proteins measured and results in normalization of the endpoint protein levels.	[26]
Ts1Cje mice	Whole brain	Control males (E14.5) *n* = 5 Ts1Cje males (E14.5) *n* = 5 control males (P0) *n* = 5 Ts1Cje males (P0) *n* = 5 control males (3 m-old) *n* = 5 Ts1Cje (three months old) *n* = 5		2D-PAGE/MALDI-TOF- MS	Calcyclin-binding protein (↑), transketolase (↑), pyruvate kinase (↑), 60S acidic ribosomal protein P0 (↑), nucleoside diphosphate kinase-B (↓)	The epression of several proteins were dysregulated in the brain of Ts1Cje mice at E14.5, but not at postnatal day 0 and 90.	[30]
Dp(10)1Yey mice *	hippocampus, cerebellum, cerebral cortex	Control females (7–9 months old) *n* = 10 Dp10 females (7–9 months old) *n* = 7 Control males (7–9 months old) *n* = 9 Dp10 males (7–9 months old) *n* = 10		Protein arrays (approximately 100 proteins/ protein modifications)	S100B (trisomic) (↑), App, Itsn, Rcan1, Pknox (in hippocampus) (↑)	The gender-specific abnormalities in the Dp10 suggest the possibility of gender-specific phenotypes in DS.	[41]
141G6 mice ** (YAC Tg)	hippocampus	Wild-type males (three months old) *n* = 10 141G6 males (three months old) *n* = 10		2D-PAGE/MALDI- TOF-MS	Electron-transfer flavoprotein α, mitochondrial (↓), NADH dehydrogenase Fe-S protein 3 (↓), NG, NG- dimethylargine dimethylaminhydrolase (↓), Flotillin-1 (↓), Profilin II (↓), Tubulin α6 (↓), Tubulin β3/4 (↑), Vimentin (↓), Hsp60 (↓), Hsp90β (↓), Peptidyl-prolyl cis-trans isomerase A (↓), 3-phosphoglycerate dehydrogenase (↑), ATP synthase α, mitochondrial (↓), Creatine kinase (↓), Fructose-bisphophate dehydrogenase (↓), Neuron specific enolase (↓), Glycerol-3-phosphate dehydrogenase (↑), Glyoxylate reductase/hydroxypyruvate reductase (↓), Guanylate kinase (↓), Isovaleryl coenzyme A dehydrogenase (↓), Phosphoglycerate kinase 1 (↓), Pyruvate kinase M2 (↓), UMP-CMP kinase (↓), Astrocytic phosphoprotein PEA-15 (↓), Dihydropyrimidinase related protein-1 (↑) -4 (↓), ES1 protein homolog, mitochondrial (↑), Protein CGI-51 homolog (↓), Heterogeneous nuclear ribonucleoprotein A2/B1/K (↓), Lamin receptor 1 (↓), ubiquitin carboxyl-terminal hydrolase isozyme L1 (UchL1) (↓), CamK2α (↓), EF-hand domain- containing protein 2 (↑), Voltage-dependent anion-selective channel protein 2 (↓)	A number of proteins were identified as molecules with altered expression in the hippocampus of 141G6 mice. In particular, a decreased level of calcium/calmodulin- dependent protein kinase type II alpha chain was identified as a candidate for cognitive impairment in DS.	[32]
152F7 mice *** (YAC Tg)	hippocampus	Wild-type males (three months old) *n* = 9; 152F7 males (three months old) *n* = 10		2D-PAGE/MALDI- TOF-MS	Fascin actin-bundling protein 1 (↑), growth factor receptor-bound protein 2 (Grb2) (↓)	Decreased Grb2 levels in the hippocampus of 152F7 mice may contribute to impaired cytoskeleton functions, and fascin dysregulation is involved in actin bundling for vesicle trafficking and may represent or lead to impaired neurotransmission.	[42]
TT2F/hChr21 mice	Neurons differentiated from ES cells	TT2F controls (in vitro differentiation day 0, 3, 6, and 10) *n* = 2; TT2F/hChr21 (in vitro differentiation day 0, 3, TOF-MS 6 and 10) *n* = 2			Calponin 3 (↓), eukaryotic translation elongation factor 1D (↓), heterogeneous nuclear ribonucleoprotein C (↓) , Hsp70 (↓), Hsp84 (↓), Hsp86-1 (↓), microtubule associated protein RP/EB family member 2 (↓), UCHL1 (↓), ubiquitin-specific-processing protease OTUB1 (↓) Annexin A4 (↑), ATPase H+ transporting V1 subunit A1 (↑), ATPase H+ transporting V1 subunit B2 (↑), Keratin 2-8 (↑), Plastin 3 (↑), Ezrin (↑)	HSA21 gene-dosage effects or chromosomal imbalance may affect the expression of cytoskeleton proteins, chaperon proteins, translation regulators, energy metabolism.	[31]

Enumerated molecules with changes of ≥25% in expression shown. * Dp(10)1Yey mice have a duplication of the Hsa21 homologous region on Mmu10, from *Prmt2* to *Pdxk*; ** 141G6: YAC encompasses four genes: *Dscr3*, *Pigp*, *Ripply3*, and *Ttc3*; *** 152F7: YAC encompasses four genes: *Dscr3*, *Dscr5*, *Ttc3*, and dual-specificity tyrosine-(Y)-phosphorylation-regulated kinase 1a; LC, liquid chromatography; ESI, electrospray ionization; MALDI, matrix-assisted laser desorption/ionization; TOF-MS, time-of-flight mass spectrometer.

**Table 3:** A comparative redox proteomic analysis of the brain with DS.

Sample	Tissue	Sample Information	Method	Differentially Expressed Proteins	Remarks	Reference
Human	frontal cortex	Young controls (12.1 ± 4.7 years old) *n* = 8 (four females, four males)	Redox proteomics (2D-PAGE/Oxyblot/MALDI-TOF-MS, Ion Trap-OrbitrapMS)	pTau(Ser404) (↑) carbonylated proteins UchL1 (↑), cathepsin D (↑), 78-kDa glucose- regulated protein (↑), V0-type proton ATPase subunit B, brain isoform (↑), glial fibrillary acidic protein (GFAP) (↑), succinyl-CoA:3-ketoacid- coenzyme A transferase 1, mitochondrial (↑)	Impairment of the proteostasis network and autophagic pathway	[33]
Human	frontal cortex	Young controls (24.9 ± 9.95 years old) *n* = 6 (two females, four males) DS (26.9 ± 17.04 years old) *n* = 6 (two females, four males) Old controls (59.2 ±7.48 years old) *n* = 6 (two females, four males) DS/AD 59.3 ± 3.44 years old) *n* = 6 (four females, two males)	Redox proteomics (2D-PAGE/4-HNE immunoblot/MALD-TOF-MS)	Protein-bound-4-hydroxy-2-nonenal (4-HNE) DS vs young control cytochrome b-c1 complex Rieske subunit, mitochondrial (↑), GFAP (↑), glutamate dehydrogenase 1, mitochondrial (↑), peroxiredoxin-2 (↑), myelin basic protein (↑), UchL1 (↑), fructose-bisphosphate aldolase-A and -C (↑), α-internexin (↑), PK isozymes M1/M2 (↑)	Impairment of several processes, including the neuronal integrity, axonal transport, synapse connections, degenerative systems, energy production, and antioxidant defense, was noted in the brains of DS and DS/AD subjects.	[43]
Ts1Cje mice	whole brain (excluding cerebellum)	Control males (3 m-old) *n* = 3 Ts1Cje males (3 m-old) *n* = 3	Redox proteomics (2D-PAGE/4-HNE and HEL immunoblot/LC-MS)	13-HPODE-bound protein ATP synthase, β chain (↑), Neuron specific enolase (↑), α-enolase (↑), Peroxiredoxin 6 (↑), Triosephosphate isomerase 1 (↑) 4-HNE-bound protein Neuron specific enolase (↑), Peroxiredoxin 6(↑), Neurofilament, light polypeptide (↑), α-internexin (↑), Phosphoglycerate kinase 1 (↑), Triosephosphate isomerase 1 (↑)	A redox proteomics approach revealed that the proteins modified with 13-HPODE and/or 4-HNE are involved in either ATP generation, the neuronal cytoskeleton, or antioxidant activity.	[34]
